# Factor structure and measurement invariance across various demographic groups and over time for the PHQ-9 in primary care patients in Spain

**DOI:** 10.1371/journal.pone.0193356

**Published:** 2018-02-23

**Authors:** César González-Blanch, Leonardo Adrián Medrano, Roger Muñoz-Navarro, Paloma Ruíz-Rodríguez, Juan Antonio Moriana, Joaquín T. Limonero, Florian Schmitz, Antonio Cano-Vindel, on behalf of the PsicAP Research Group

**Affiliations:** 1 Mental Health Centre, University Hospital “Marqués de Valdecilla”- IDIVAL, Santander, Spain; 2 Faculty of Psychology, University Siglo 21, Córdoba, Argentina; 3 Department of Basic Psychology, Faculty of Psychology, University of Valencia, Valencia, Spain; 4 Castilla La Nueva Primary Care Centre, Health Service of Madrid, Madrid, Spain; 5 Department of Psychology, University of Córdoba/ Maimónides Institute for Research in Biomedicine of Cordoba-IMIBIC/Reina Sofía University Hospital, Córdoba, Spain; 6 Department of Basic Psychology, Autonomous University of Barcelona, Bellaterra, Barcelona, Spain; 7 Department of Psychology, Ulm University, Ulm, Germany; 8 Department of Basic Psychology, University Complutense of Madrid, Madrid, Spain; King's College London, UNITED KINGDOM

## Abstract

The Patient Health Questionnaire (PHQ-9) is a widely-used screening tool for depression in primary care settings. The purpose of the present study is to identify the factor structure of the PHQ-9 and to examine the measurement invariance of this instrument across different sociodemographic groups and over time in a sample of primary care patients in Spain. Data came from 836 primary care patients enrolled in a randomized controlled trial (PsicAP study) and a subsample of 218 patients who participated in a follow-up assessment at 3 months. Confirmatory factor analysis (CFA) was used to test one- and two-factor structures identified in previous studies. Analyses of multiple-group invariance were conducted to determine the extent to which the factor structure is comparable across various demographic groups (i.e., gender, age, marital status, level of education, and employment situation) and over time. Both one-factor and two-factor re-specified models met all the pre-established fit criteria. However, because the factors identified in the two-factor model were highly correlated (*r* = .86), the one-factor model was preferred for its parsimony. Multi-group CFA indicated measurement invariance across different demographic groups and across time. The present findings suggest that physicians in Spain can use the PHQ-9 to obtain a global score for depression severity in different demographic groups and to reliably monitor changes over time in the primary care setting.

## Introduction

Depression is one of the most common mental disorders around the world. Globally, the total number of people with depression was estimated to exceed 300 million in 2015 [1]. Depression is responsible for more ‘years lost’ to disability than any other condition in the world (according to WHO estimates, 7.5% of all years lived with disability in 2015), and it is a major contributor to the overall global burden of disease [1]. Depression is ranked by WHO as the single largest contributor to global disability. The lifetime prevalence for major depressive disorder (MDD) has been estimated at 12.8% in Europe (ESEMeD project) [2] and 10.6% in Spain [3]. The Diagnostic and Assessment Study of Mental Disorders in Primary Care (DASMAP), based on 3,815 patients from 77 primary care centres in Spain, found that almost 30% reported a lifetime history of MDD, with 9.6% experiencing MDD in the past 12 months [4]. Nevertheless, epidemiological and clinical studies show that general practitioners (GP) fail to diagnose a large part of patients suffering from depression [5]. In fact, studies conducted in Spanish primary care settings show that nearly 78% of the patients with depression are misdiagnosed, indicating that only approximately one-quarter of cases diagnosed as depressed by GPs are correctly diagnosed [6]. Moreover, a high proportion of these individuals remain untreated [7]: in Spain, only approximately one-third of patients with MDD receive "minimally-adequate" treatment [8].

The Patient Health Questionnaire Depression Scale (PHQ-9) [9] is well-validated instrument used to quickly diagnose depression, assess severity, and monitor treatment response. The PHQ-9 includes the nine signs/symptoms for the diagnosis of MDD in the Diagnostic and Statistical Manual of Mental Disorders, Fourth Edition (DSM-IV) and it is a widely-used measure to assess depression in various different settings [10], particularly in primary care [11] where most patients with depression are first diagnosed and treated [12].

The Spanish version of the PHQ-9 is a reliable and valid measure of depression in hospitalized patients [13] and in primary care patients [14] in Spain. However, no studies have yet examined the underlying factor structure of this instrument in the Spanish population. Studies conducted in other Spanish-speaking populations have reported mixed results that support both a one-factor structure [15–18] as well as a two-factor structure that includes both affective and somatic symptoms [19, 20]. For instance, Huang et al [17] investigated the factor structure of the PHQ-9 in a large sample of 5,053 primary care patients, finding that one general factor accounted for the communality of all PHQ-9 items. Additionally, a Spanish version of the scale developed in Mexico presented a unidimensional factor structure in a large sample (n = 55,555) of Mexican women [16]. Some studies in different countries and settings also support a unidimensional structure [21, 22], although other studies have identified a two-factor solution [23, 24].

When validating a scale such as the PHQ-9, it is important to examine whether that scale remains structurally stable over time and across different groups (i.e., measurement invariance). Examination (and confirmation) of factorial invariance is critical to assure comparability of clinical outcomes. If invariance is not given, the interpretation of manifest scores (such as feeling down or little pleasure in doing things) does not necessarily reflect the same latent factor (i.e., depression) in different groups or at different times [25]. With regard to the PHQ-9, little research has been conducted to determine whether this scale is invariant across various demographic groups (other than gender) or over time. Petersen et al. [24] found that the PHQ-9 was invariant in a sample of male and female primary care patients, and Chilcot et al. [26] showed that the factor structure of this scale was invariant over time within a palliative care population. To the best of our knowledge, no studies have yet investigated the invariance of the PHQ-9 factor structure in any Spanish version of this instrument.

Therefore, the present study had two primary aims. The first aim was to identify the factor structure of the PHQ-9 in a sample of primary care patients in Spain. The second aim was to examine the invariance of structural properties across various sociodemographic groups (i.e., gender, age, marital status, level of education, and employment situation) and over time.

## Method

### Patients

This study was conducted at 22 primary care centres included in the Psychology in Primary Care (PsicAP) study [27] between the months of January and July (inclusive), 2016. The PsicAP study is a randomized controlled trial developed to test the effectiveness of a group-delivered transdiagnostic cognitive behavioral therapy (TD-CBT) versus treatment as usual (TAU) in the treatment of emotional disorders in primary care settings in Spain. During the study inclusion period, all patients aged 18 to 65 years who consulted with their GP at any of these 22 PsicAP centers and who presented a diagnosis or suspected diagnosis of an emotional disorder (anxiety, depression or somatization disorder) were invited to participate in the clinical trial. A total of 836 participants agreed to participate and were therefore included in the baseline sample used to study the factor structure of the PHQ-9. Of these 836 patients, a subgroup of 218 participants (who were finally included in the RCT and re-evaluated with the PHQ-9 three months later) was used to assess factorial invariance over time. At first contact, all candidates were given a patient information sheet containing full details about the study purpose. Subjects were required to sign an informed consent form to participate. The socio-demographics of the participants are shown in Table 1.

**Table 1:** Demographics characteristics of sample.

	Total sample		Follow-up subsample	
	(*N* = 836)		(*n* = 218)	
	*N*	%	n	%
**Gender**				
Female	639	76.4	172	78.9
Male	197	23.5	46	21.1
**Age group**				
≤ 19	10	1.2	1	0.5
20–39	308	36.8	76	34.9
40–59	438	52.4	120	55.0
≥ 60	80	9.6	21	9.6
**Marital status**				
Married	411	49.2	130	59.6
Divorced	77	9.2	12	5.5
Widowed	24	2.9	8	3.7
Separated	51	6.1	5	2.3
Never married	162	19.4	37	17
Unmarried	111	13.1	26	11.9
**Level of education**				
No schooling	15	1.8	0	0
Basic education	248	29.7	49	22.5
Secondary education	165	19.6	47	21.6
High School	170	20.4	53	24.3
Bachelor	197	23.5	60	27.5
Master/doctorate	41	5.0	9	4.1
**Employment situation**				
Part-time employee	119	14.2	31	14.2
Employed full time	308	36.8	77	35.5
Unemployed, in search of work	201	24.0	53	24.3
Unemployed, not looking for work	107	12.9	27	12.4
Temporary incapacity to work	44	5.3	10	4.6
Permanent incapacity to work	12	1.4	5	2.3
Retired	45	5.4	15	6.9

A total of 218 participants completed the PHQ-9 again 3 months after the initial baseline assessment. The demographic characteristics of this smaller subsample was similar to the original sample, with no significant differences in gender distribution (χ^2^ = 0.55, *df* = 1, *p* = .46), age (t = 1.18, *df* = 1051, *p* = .24), or employment condition (χ^2^ = 1.95, *df* = 6, *p* = .92) (see Table 1). However, significant differences were observed in the distribution of marital status (χ^2^ = 11.75, *df* = 5, *p* = .03) and level of education (χ^2^ = 12.74, *df* = 5, *p* = .03). Given the actually large sample size and the sensitivity of the χ^2^ statistic to sample size, these differences should not be over-interpreted. In fact, the effect size of these differences were considerably low (Cramer’s V level of education = .10; Cramer’s V Marital Status = .11). We further compared whether missing cases significantly differed from non-missing cases on each PHQ-9 item. We found statistically significant differences (without adjusting for multiple comparisons) in item 1 (loss of interest) [t (834) = 2.48, p = 0.01], and item 9 (suicidal ideation) [t (834) = 2.20, p = 0.03]. However, the effect sizes for these differences were small (Hedges' g < 0.2) and thus unlikely to affect the results.

### Measures

*Patient Health Questionnaire-9 (PHQ-9) [9].* The PHQ-9 is part of the PHQ and consists of nine items designed to assess the nine DSM-IV diagnostic criteria for MDD. The scales check for the presence of the following symptoms over the previous two weeks: (a) depressed mood; (b) anhedonia; (c) sleep problems; (d) feelings of tiredness; (e) changes in appetite or weight; (f) feelings of guilt or worthlessness; (g) difficulty concentrating; (h) feelings of sluggishness or worry; and (i) suicidal ideation. Items are answered on a four-point Likert scale from 0–3 as follows: 0 (never), 1 (several days), 2 (more than half of the days), and 3 (most days). The Spanish version of the PHQ-9 was used in the present study. This version has demonstrated good psychometric properties, as follows: internal consistency, McDonald’s ω = .89; 88% sensitivity; 80% specificity; and positive and negative predictive values of 92% and 72%, respectively [14].

### Procedure

GPs at the participating centers were asked to identify patients who presented signs or symptoms of anxiety, depression, or physical symptoms for which no biological cause could be found. The GP then asked these candidates to participate in the study. Patients who agreed then signed the informed consent form and were scheduled to meet with a clinical psychologist, who again provided the patients with verbal and written details about the study to be sure they fully understood it. At this same appointment, the particpants were asked to complete a computer-based version of the PHQ and the other study measures (n = 836). Patients with impaired vision received assistance in completing the questionnaires. Paper versions of these instruments were provided to patients who had difficulties using the computer.

A subsample of 218 cases was assessed at the 3-month follow-up. This subsample received one of the two treatments tested in the randomized controlled trial: (i) TAU, mainly pharmacological treatment by the GP, or (ii) seven 90-minute group sessions of TD-CBT delivered over 12 to 14 weeks (for further details see [27]).

### Ethical aspects

The study was conducted in accordance with the Declaration of Helsinki. This project is supported by the Psicofundación (Spanish Foundation for the Promotion, Scientific and Professional Development of Psychology) and approved by the Corporate Clinical Research Ethics Committee of primary care of Valencia (CEIC-APCV), Spain, as the national research ethics committee coordinator, and the Spanish Agency of Medicines and Medical Devices (AEMPS) (EUDRACT: 2013-001955-11 and Protocol Code: ISRCTN58437086).

The study was conducted in accordance with the Spanish Law on Data Protection. Patient participation in the study was voluntary and participants were able to withdraw at any time without explanation and without negative consequences for future medical care.

### Statistical analysis

Initially, an exploratory analysis of the data was performed to explore the behaviour of the variables, to evaluate the quality of the data, and to check that requirements of statistical tests were met. We checked for the existence of atypical cases, missing values and compliance with the statistical assumptions (linearity, and univariate and multivariate normality). A confirmatory factor analysis (CFA) was performed using maximum-likelihood (ML) as an estimation method; the AMOS 20 program [28] was used for these analyses. A one-factor model was specified in which the nine PHQ-9 items loaded on a single factor (called "depression") and an alternative two-factor model in which 3 items loaded on the "somatic" factor and 6 items in the "cognitive-affective" factor, corresponding with previous research on PHQ structure.

Multiple indices were examined to determine model fit: (a) The chi-squared statistic (χ^2^) was reported following conventions. However, given its sensitivity to sample size, is becomes usually significant. However, differences in χ^2^ can be interpreted to compare nested models. Additionally, we considered (b) the comparative fit index (CFI); (c) the Tucker-Lewis index (TLI); and (d) the root mean square error of approximation (RMSEA). To interpret these indices, we used the critical values previously recommended [29, 30]. Specifically, values > .90 and .95 for the CFI and TLI were considered benchmarks for acceptable and good fit, respectively; and RMSEA values of < .08 and .06 were benchmarks for acceptable and good fit, respectively.

Analyses of multiple-group invariance were conducted to determine the extent to which the factor structure was comparable across various sociodemographic groups (i.e., gender, age, marital status, level of education and employment situation) and over time. We followed the measurement invariance procedures outlined by Brown [31]. Factorial invariance is essential to provide meaningful comparisons of scores across groups or across time. Therefore, four levels of measurement invariance were sequentially tested (configural, weak, strong, and strict invariance), where each level introduces more equality constraints across groups. Configural invariance implies that the pattern of fixed and free factor loadings are equivalent. Weak factorial invariance examines the equivalence of factor loadings (i.e., items assess the latent variable in the same way across groups or time). Strong factorial invariance examines the equality of latent means, implying that any differences in means on the scale are due to true differences in means across groups or time. Finally, strict invariance—the most restrictive level of factorial invariance—examines the invariant item residual variances and this implies that group differences in variances of scale scores are due only to group differences in depression variances, since error variances were constant across groups. Configural invariance is supported if the same unconstrained factor structure simultaneously fit for the split groups yields a good fit. After testing configural invariance, we examined weak/metric invariance. The fit of the restricted model (equal factor loadings across groups) and the free model, were compared in terms of their χ^2^ values. A non-significant increase in the χ^2^ value (relative to *df*) in the constrained model relative to the unconstrained model indicated that the constrains across groups were possible. As an additional criterion, the change in the CFI coefficient was considered. If the drop in CFI of the constrained model relative to the unconstrained model did not exceed 0.01, the constrained model was accepted [32]. The ∆CFI criterion was argued to be superior to ∆ χ^2^, as it is less sensitive to sample size [33]. We proceeded analogously to tests strong/scalar invariance and strict invariance.

## Results

### Exploratory and descriptive analysis

The *Z* scores for each item were calculated and univariate values considered atypical were those values outside the *Z* ± 3 range [34]. The existence of atypical multivariate cases was assessed using the Mahalanobis distance (D^2^) statistical procedure. There were no univariate atypical cases and only 7 cases showed a statistically significant distance from the centroid of the group (p < .001). In the analysis of missing values, the items presented < 5% of lost cases. Considering the low proportion of missing treatment values, "listwise deletion" was applied in accordance with published recommendations [35].

Based on the criteria proposed by George and Mallery [34], all items presented a distribution that was close to normal given that asymmetry and kurtosis values were between ±2 (see Table 2). Multivariate normality showed a Mardia index of 7.24, indicating there was no substantial deviation from normal distribution. To test the linearity assumption of the relations, linear and curvilinear estimates were calculated between pairs of items. In all cases, the linear function was superior to the curvilinear function, thus confirming the linearity assumption. S1 Table shows the inter-item correlation matrix for the PHQ-9 items.

**Table 2:** Descriptive statistics of the PHQ-9 items.

	M	SD	Skewness	Kurtosis
1. Little interest or pleasure	1.70	1.01	- 0.05	- 1.20
2. Feeling down, depressed, or hopeless	1.81	0.99	- 0.13	- 1.21
3. Trouble falling/staying asleep/sleeping too much	1.75	1.10	- 0.23	- 1.30
4. Feeling tired or having little energy	1.86	1.03	- 0.30	- 1.20
5. Poor appetite or overeating	1.52	1.12	0.00	- 1.36
6. Feeling bad about yourself/failure	1.57	1.16	- 0.02	- 1.46
7. Trouble concentrating	1.36	1.07	0.26	- 1.18
8. Moving or speaking so slowly	1.21	1.03	0.40	- 1.00
9. Thoughts that you would be better off dead	0.61	0.90	1.46	1.18

### Confirmatory factor analysis

**Fig 1:**
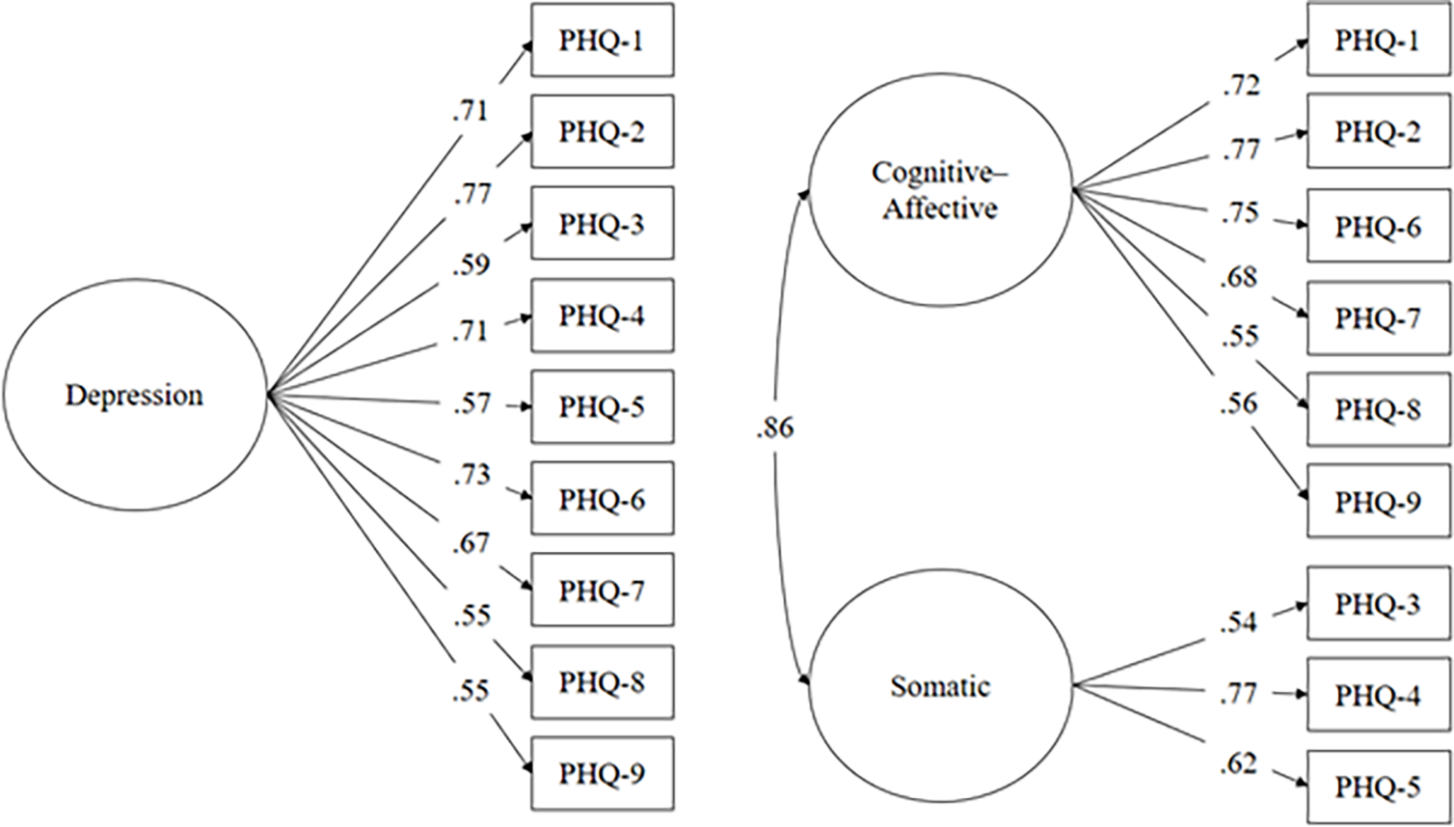
One-factor and two-factor models of the PHQ-9 items.

Since normality assumptions were met, the maximum-likelihood (ML) was used as an estimation method. Both the one-factor and two-factor models presented acceptable values for the CFI and GFI indexes, but exceeded an RMSEA of .08. The model fit could be substantially improved when error terms of PHQ-9 items 1 and 2 were allowed to be correlated, reflecting that both items shared similarity that is not explained by the general depression factor. The fit indexes obtained for both models are summarized in Table 3. All items displayed substantial factor saturation, as indicated by their high factor loading (all λ .54-.77; all *p*< 0.05; see Fig 1). The two-factor model yielded a better fit in both the original model and the models that additionally allowed for the error correlations of PHQ items 1 and 2. However, the "somatic" and "cognitive-affective" factors were found to be highly correlated (*r* = .86). This indicates substantial overlap between the two factors and complicates the interpretation of corresponding test scores for diagnostic purposes.

**Table 3:** Model fit.

	Fit indexes					
	χ^2^	*df*	*p*	CFI	GFI	RMSEA
M1: One factor	286,03	27	.00	.91	.92	.10
M2: Two factors	213,12	26	.00	.94	.94	.09
M3: One factor*	182,15	26	.00	.95	.95	.08
M4: Two factors*	126,73	25	.00	.96	.97	.07

* Error terms of PHQ item 1 and 2 were allowed to be correlated.

### Invariance across sociodemographic groups

Given the known issues of using the ML estimation method with ordinal data, we used the ML and the Weighted Least-Squares (WLS) methods to perform the CFA. The results obtained with both estimation methods (ML and WLS) were highly similar and therefore we report only the results of the ML estimation. To determine whether patient gender affected the measurement model, the sample was split into men (23.6%) and women (76.4%), and constrains were introduced to test if parameters could be constrained across both groups. Then, we compared the fit of the constrained model to that of the free model were parameters were estimated independently in each group. Both models were compared in terms of ∆χ^2^ and, more importantly, by ∆CFI: The more constrained model was accepted if the constraints did not significantly deteriorate model fit [29, 32]. Following Brown [31], we first examined the fit of the single-sample one-factor solutions within the specific subsamples (e.g., men-only and women-only subsample) separately. Next, we tested four levels of invariance (configural invariance, weak invariance, strong invariance, and strict invariance) using a series of increasingly restrictive models. The single-sample solutions for the one-factor model based on the men-only and women-only subsamples are shown in Table 4. Both subsamples were associated with good model fit. The test of configural invariance was supported, as evidenced by fit indices meeting the benchmarks for adequate fit (RMSEA = .058, CFI = .95, TLI = .93). All levels of invariance up to strict invariance could be assumed across gender, as evidenced by a non-significant drop in model fit (∆CFI < .01) for the successively more constrained models (Table 4).

**Table 4:** Fit statistics for multi-group confirmatory factor analysis by gender, age, marital status, level of education, and employment situation.

	χ^2^	Df	RMSEA	CFI	TLI	ΔCFI	Δ χ^2^	Δ df
**Gender**								
Women	128.77	26	.079	0.95	0.93	—	—	—
Men	68.40	26	.070	0.95	0.93	—	—	—
Configural Invariance	197.26	52	.058	0.95	0.93	—	—	—
Weak Invariance	204.14	60	.054	0.95	0.94	0.00	6.88	8
Strong Invariance	262.71	69	.058	0.94	0.93	0.01	58.57**	9
Strict Invariance	280.67	79	.056	0.94	0.94	0.00	17.96	10
**Age**								
Young adults (20–39)	102.93	26	.087	0.93	0.90	—	—	—
Adults (40–59)	107.24	26	.085	0.95	0.93	—	—	—
Configural Invariance	210.19	52	.064	0.94	0.92	—	—	—
Weak Invariance	214.48	60	.059	0.94	0.93	0.00	4.29	8
Strong Invariance	226.82	69	.055	0.94	0.94	0.00	12.34	9
Strict Invariance	246.18	79	.053	0.94	0.94	0.00	19.36	10
**Marital Status**								
Paired	132.40	26	.080	0.94	0.92	—	—	—
Unpaired	76.65	26	.079	0.95	0.94	—	—	—
Configural Invariance	209.05	52	.060	0.95	0.93	—	—	—
Weak Invariance	218.68	60	.057	0.95	0.94	0.00	9.63	8
Strong Invariance	245.92	69	.056	0.94	0.94	0.01	27.24**	9
Strict Invariance	263.18	79	.053	0.94	0.94	0.00	17.26	10
**Level of Education**								
Basic education	49.16	26	.060	0.97	0.96	—	—	—
Secondary education	116.53	26	.088	0.93	0.90	—	—	—
High education	54.82	26	.073	0.96	0.95	—	—	—
Configural Invariance	220.49	78	.047	0.95	0.93	—	—	—
Weak Invariance	244.22	94	.044	0.95	0.94	0.00	23.73	16
Strong Invariance	293.04	112	.045	0.94	0.94	0.01	48.82**	18
Strict Invariance	334.64	132	.044	0.93	0.94	0.01	41.60**	20
**Employment situation**								
Employed	111.94	26	.089	0.94	0.92	—	—	—
Unemployed	112.51	26	.090	0.94	0.92	—	—	—
Configural Invariance	224.46	52	.063	0.94	0.92	—	—	—
Weak Invariance	244.62	60	.061	0.94	0.93	0.00	20.16**	8
Strong Invariance	271.46	69	.600	0.93	0.93	0.01	26.84**	9
Strict Invariance	276.24	79	.055	0.93	0.94	0.00	4.78	10

**p<0.01

Using the same procedure, the model was evaluated to check for invariance across age (Table 4). To this end, participants were divided into a younger adult group (20 to 39 years of age) and an older adult group (40 to 59 years old) following the criteria recommended by Martín Ruiz [36]. Adolescents (< 20 years) and elderly (> 60 years) were not included in this analysis because they did not reach the minimum group sample size (> 200 cases) [37]. After dividing patients into younger adults (*n* = 316) and older adults (*n* = 433), successively stricter constrains were tested to test for configural, weak, strong, and strict invariance. The single-sample solutions for the one-factor model based on the *younger adult group* and the *older adult group* subsamples are shown in Table 4. A good model fit was obtained for both subsamples. Configural invariance was supported by fit indices meeting benchmarks for adequate fit (RMSEA = .064, CFI = .94, TLI = .92). Weak, strong, and strict invariance could be assumed across age groups, as evidenced by a non-significant drop in model fit (Δχ^2^ = *n*.*s*. and ∆CFI < .01) for the successively stricter models (Table 4).

To evaluate invariance across different groups of marital status, participants were split into a "*paired group*" (married and cohabiting participants; *n* = 522) and "*unpaired group*” *(*divorced, separated, widowed, and unmarried; *n* = 314). Successively stricter constrains were tested to evaluate configural, weak, strong, and strict invariance. The single-sample solutions for the one-factor model based on the *paired group* and *unpaired group* subsamples are shown in Table 4, each showing a good model fit. Configural invariance was supported by fit indices meeting benchmarks for adequate fit (RMSEA = .063, CFI = .94, TLI = .92). Weak, strong, and strict invariance could be assumed across pairing status, as evidenced by a non-significant drop in model fit for the stricter models (∆CFI < .01) (Table 4).

To evaluate invariance across levels of education, participants were split into three groups: *basic education group (n = 248); secondary education group (n = 335) and high education (n = 238)*. Successively stricter constrains were tested to evaluate configural, weak, strong, and strict invariance. Configural invariance was supported by fit indices meeting the benchmarks for adequate fit (RMSEA = .047, CFI = .95, TLI = .93). Weak, strong, and strict invariance could be assumed across educational levels, as evidenced by a non-significant drop in model fit for the stricter models (∆CFI < .01) (Table 4).

To test invariance across employment conditions, participants were divided into two groups, an *employed group* (full and part-time employment; *n* = 422), and an *unemployed group* (unemployed, incapacity to work, and retired; *n* = 408). Successively stricter constrains were tested across groups to evaluate configural, weak, strong, and strict invariance. Model fits obtained for the *employed subsample* and for the *unemployed group* subsample are shown in Table 4. These were good for both subsamples. Configural invariance was supported by fit indices meeting the benchmarks for adequate fit (RMSEA = .063, CFI = .94, TLI = .92). Weak, strong, and strict invariance could be assumed across employment conditions, as evidenced by a non-significant drop in model fit for the stricter models (∆CFI < .01) (Table 4).

### Longitudinal invariance

Temporal stability, also known as longitudinal invariance, is important to make sure the instrument in question measures the same latent constructs in the same manner over time. Using an analogous procedure, we evaluated whether the measurement model was invariant across time (i.e., a 3-month interval from baseline to the 3-month assessment). Applying successively stricter parameter constrains, the analyses supported weak (∆χ^2^ = 60.70, *df* = 8, *p* = .57; ∆CFI < .01), strong (∆χ^2^ = 117.87, *df* = 17, *p* < .01; ∆CFI < .01), and strict invariance (∆χ^2^ = 117.87, *df* = 26, *p* < .01; ∆CFI < .01) across time.

## Discussion

To our knowledge, this is the first study to examine the factor structure of the Spanish version of the PHQ-9 in the Spanish population. The results of the confirmatory factor analyses in the data collected from this primary care sample identified one-factor and two-factor models, both of which met all the pre-established fit criteria. However, given that the "somatic" and "cognitive-affective" factors identified in the two-factor model were highly correlated (*r* = .86), the unidimensional model is more parsimonious and, hence, the preferred solution. Importantly, this one-factorial structure was found to be invariant across various demographic groups, including gender, age, marital status, level of education, and employment situation. Thus, the PHQ-9 is applicable in the Spanish population and derived scores can be validly compared without need for specific sociodemographic adjustments. Additionally, the one-factor structure was found to be stable over a 3-month period.

Consistent with the results reported in previous studies of Mexican females [16] and a US-based Spanish-speaking Latino population [15, 17, 18], our findings support the one-factor solution for the Spanish version of the PHQ-9. Most studies examining the factor structure of the PHQ-9 have corroborated the unidimensionality of the scale [21, 22, 38–40]. However, some authors have reported a two-factor structure comprising a cognitive–affective and a somatic dimension [23, 24, 26, 41, 42]. This discrepancy between studies is likely due to differences in patient populations. The current study mainly consisted of individuals with mild to moderate emotional distress in a primary care setting, which is the kind of heterogeneous sample for which the PHQ-9 was originally developed and validated to diagnose depression [9]. By contrast, studies that have found a two-factor solution have been conducted in populations that predominately present comorbid physical conditions such as spinal cord injury [42] or cancer [26, 41]; therefore, somatic factor loading may be attributable to possible confounding effects of the physical illness [43].

Petersen et al [24] found that a two-factor model with five ‘somatic’ items and four ‘affective/non-somatic’ items yielded the best fit in a sample of primary care patients. The one-factor and two-factor structures examined in our study displayed a poor model fit in the study carried out by Petersen et al [24]. However, it is important to note that all the patients in their sample had a diagnosis of major depression (PHQ-9 >9) for which antidepressive treatment was indicated. As those authors suggested, their relatively homogeneous sample may have resulted in range restriction in the measures, thereby attenuating correlations among variables. When PHQ-9 is used with more heterogeneous samples, it is more likely to produce a one-factor solution because the variance is larger and therefore the items are more likely to load on one factor [24]. Alternatively, it may well be that somatic features are more relevant in the diagnosis of depression in samples that include individuals with moderate to severe clinical conditions. It is worth noting that while the one-factor model seems to be a more parsimonious solution in our study, the two-factor model (with a factor of 3 ‘somatic’ items) also displayed a decent model fit.

Gender invariance, which may be considered a prerequisite for making quantitative comparisons, adds important support for the validity of the PHQ-9 as a self-report screening instrument because it indicates that the measurement model of the latent depression construct is comparable in both sexes. This implies that differences in observed test scores between men and women reflect true differences in depression rather than an artefact of the measurement method. Epidemiological studies have consistently shown a higher prevalence of MDD in women than in men [44, 45]. In Spain, the ESEMED study found 12-month prevalence rates for MDD of 2.2% for males and 5.6% for females. Moreover, this difference was even more pronounced for "any depressive disorder"; 2.3% for males vs. 6.3% for females [3]. Although we found a statically significant difference between men and women in mean PHQ-9 scores, the effect size was small.

Apart from gender, we also tested invariance across other sociodemographic characteristics such as age, marital status, level of education, and employment situation. Invariance could be assumed for all of these variables when patients were divided into comparable groups according to the criteria recommended by Martín Ruiz [36] and Barret [37]. These results strengthen the validity of the PHQ-9 as a screening tool in settings (such as primary care) where population heterogeneity is substantial.

To precisely measure the true change and inter-individual differences, it is also critical to examine if the PHQ-9 consistently measures the same construct over time. Previous studies have reported mixed results for invariance across time. One study [26] found that the PHQ-9 was invariant over time in a sample of patients newly referred to a palliative care service. In that study, patients were assessed within one-week of referral (time 1) and then again 4-weeks later (time 2). The observed factor structure of the PHQ-9 appeared to be stable in this setting since the two-factor model had good fit at both time points. By contrast, other studies in patients with spinal cord injuries have reported considerable instability of the factor structure over time [42, 43]. In our results, the factor structure was equivalent at two different time points (baseline and at 3 months) during which patients received pharmacological and/or psychological treatment for emotional disorders in a primary care setting [27]. This invariance indicates that changes in PHQ-9 scores over time reflect true changes in the underlying latent construct (i.e. depression) rather changes in the assessment or structure of the scale. The effect size for this change was large (g = 0.78) and we believe this change can be reasonably attributed to the effects of treatment.

### Study strengths and limitations

The main strengths of the present study is the relatively large, heterogeneous sample of patients with emotional disorders from 22 primary care centres across Spain. It is worth noting that in Spain, as in many other countries, the vast majority of patients with emotional disorders are diagnosed and treated only in the primary care setting [46]. Thus, our sample can be considered representative of persons with mild-to-moderate emotional disorders seeking help. One limitation of our study is that the PHQ-9 may have a different structure in individuals in need of specialized services, or those with more severe clinical conditions; and this possibility could not be tested in the current study. We did not assess the concurrent validity of the Spanish version of the PHQ-9 against a semi-structured clinical interview to establish empirically-derived cutoff levels. However, the good sensitivity and specificity of the PHQ-9 have already been demonstrated in different populations [47], including the Spanish cultural context [13, 14]. The subsample of patients assessed at follow-up was part of an ongoing RCT to compare group CBT to TAU in a primary care setting. As a result, the subsample assessed at follow-up may not have accurately reflected the overall sample assessed at baseline. Indeed, some small but significant differences were observed in terms of marital status and level of education between baseline and follow up assessments. We also found small but statically significant differences between the baseline sample and the subsample used to assess factorial invariance over time on two items of the PHQ-9. Although these differences were small (Hedges' g < 0.2) and significance was not adjusted for multiple comparisons, the findings obtained from this subsample (i.e., the results of invariance over time) need to be interpreted with caution. Finally, a potential limitation regarding the data used to calculate invariance over time is that these data came from a RCT, which implies an intervention between two time-points (i.e., baseline and assessment). However, most patients who seek treatment for psychological distress in primary care will receive some kind of pharmacological or psychological treatment from their GP, and thus the fact that invariance over time was based on a subsample of patients included in a RCT (and, therefore, who received some type of intervention) may actually reflect the real-world context in which PHQ-9 is likely to be used. Thus, it might be said that the PHQ-9 structure is invariant regardless of treatment for emotional disorders. Indeed, the fact that strict invariance holds provides additional support for the robustness of the latent structure of the scale.

### Clinical and research implications

Our findings have several important clinical and research implications. First, given the high prevalence rates of emotional disorders and, particularly, depression among primary care patients, GPs need well-validated screening tools that are easy to administer, score, and interpret. The unidimensional factor structure of the PHQ-9 allows clinicians to use the scale without the need for complicated scoring algorithms. Thus, clinicians and researchers can rely on a global score and a single cut-off score. Second, measurement invariance across multiple groups provides empirical support to allow clinicians and researchers to interpret between-group comparisons as true differences in depression intensity and not a measurement artifact. For instance, establishing gender invariance (or non-invariance) is critical for studies with a gender perspective. Finally, invariance measurement across time further supports the utility of the PHQ-9 as a simple tool for monitoring treatment response.

### Conclusions

The current study provides important evidence regarding the construct validity and multi-group factorial and time invariance of the Spanish version of the PHQ-9 in primary care settings in Spain. In the present study, both the one-factor and the two-factor models displayed good model fit. The two-factor model (with a factor of three ‘somatic’ items highly correlated to the ‘cognitive-affective’ factor) displayed slightly better fit, while the one-factor model was preferred for its parsimony. The one-factor model was additionally demonstrated to be invariant across demographic groups and across time. The findings presented here suggest that primary care physicians in Spain can use the PHQ-9 to assess depression severity and to reliably monitor changes over time.

## Supporting information

S1 Table PHQ-9 inter-item correlation matrix. (DOCX)
