# Supplementary material for: Factor structure and measurement invariance across various demographic groups and over time for the PHQ-9 in primary care patients in Spain
Source: PLoS One. 2018 Feb 23;13(2):e0193356. doi: 10.1371/journal.pone.0193356 (PMC5825085; doi:10.1371/journal.pone.0193356)
Supplement: S1 Table — (DOCX) [file pone.0193356.s001.docx]

| **Table S1- PHQ-9 Inter-item correlation matrix** | | | | | | | | | |
| --- | --- | --- | --- | --- | --- | --- | --- | --- | --- |
| items | 1 | 2 | 3 | 4 | 5 | 6 | 7 | 8 | 9 |
| 1 | 1.000 | .742 | .411 | .554 | .365 | .544 | .479 | .315 | .389 |
| 2 | .742 | 1.000 | .453 | .577 | .382 | .591 | .499 | .371 | .434 |
| 3 | .411 | .453 | 1.000 | .487 | .410 | .377 | .344 | .338 | .293 |
| 4 | .554 | .577 | .487 | 1.000 | .472 | .436 | .440 | .347 | .320 |
| 5 | .365 | .382 | .410 | .472 | 1.000 | .400 | .335 | .386 | .283 |
| 6 | .544 | .591 | .377 | .436 | .400 | 1.000 | .520 | .406 | .475 |
| 7 | .479 | .499 | .344 | .440 | .335 | .520 | 1.000 | .472 | .363 |
| 8 | .315 | .371 | .338 | .347 | .386 | .406 | .472 | 1.000 | .321 |
| 9 | .389 | .434 | .293 | .320 | .283 | .475 | .363 | .321 | 1.000 |
